# First‐Line Levofloxacin‐Based Triple Therapy Versus Standard Bismuth‐Based Quadruple Therapy for *Helicobacter pylori* Eradication in Saudi Arabia: A Retrospective Single‐Center Study

**DOI:** 10.1002/hsr2.70432

**Published:** 2025-02-05

**Authors:** Abdulrhman Khaled Al Abdulqader, Turki Abdullah Alamri, Mahdi Abdullah Alhamad, Somaia Shehab El‐Deen, Abdallah Essa, Raed Abdullah Alfayez, Baqer Mohammed Albaqshi, Adnan Salah Almajed, Mohammed Yousef Alhassan, Ali Essa, Ahmed Abdullah Albadrani, Omar Alomair, Bashaeer Abdullh Al Jalal, Mohammed Yousef Almulhim, Abdullah Alotaibi, Ehab Darwish

**Affiliations:** ^1^ Internal Medicine Department College of Medicine, King Faisal University Al‐Ahsa Kingdom of Saudi Arabia; ^2^ Internal Medicine Department, Gastroenterology Unit King Fahad University Hospital Al Khobar Kingdom of Saudi Arabia; ^3^ Imam Abdulrahman Bin Faisal University Dammam Kingdom of Saudi Arabia; ^4^ King Fahad Specialist Hospital Dammam Kingdom of Saudi Arabia; ^5^ Tropical Medicine Department, Faculty of Medicine Menoufia University Shebin El‐Kom Egypt; ^6^ King Fahad Medical City Riyadh Kingdom of Saudi Arabia; ^7^ King Saud Medical City Riyadh Kingdom of Saudi Arabia; ^8^ College of Medicine, King Faisal University Al-Ahsa Kingdom of Saudi Arabia; ^9^ Faculty of Medicine, Menoufia University Shebin El‐Kom Egypt; ^10^ Internal Medicine Department College of Medicine, Prince Sattam Bin Abdulaziz University Al‐Kharj Kingdom of Saudi Arabia; ^11^ Hepatology, Gastroenterology and Infectious Diseases Department, Faculty of Medicine Zagazig University Zagazig Egypt

**Keywords:** bismuth, efficacy, eradication, *H. pylori*, levofloxacin

## Abstract

**Background and Aims:**

Antibiotic resistance in Saudi Arabia has led to decreased efficacy of conventional triple therapy for *Helicobacter pylori* (*H. pylori*) eradication, prompting the development of alternative treatments like levofloxacin‐based triple and bismuth‐based quadruple therapies. However, comparative data regarding its efficacy are lacking. Therefore, this study's goal was to compare the efficacy of levofloxacin‐based triple therapy with that of standard bismuth‐based quadruple therapy as first‐line regimens.

**Methods:**

This retrospective analysis included 197 treatment‐naïve adults with *H. pylori* infection who received levofloxacin‐based triple (levofloxacin + amoxicillin + PPI) therapy (*n* = 81) or standard bismuth‐based quadruple (bismuth + tetracycline + metronidazole + PPI) therapy (*n* = 116). *H. pylori* eradication was evaluated 4–8 weeks after medication administration using the ^13^C‐urea breath test, and variables that could affect the rate of success were examined.

**Results:**

There were no differences between groups in terms of age, sex, nationality, or type of proton pump inhibitor (PPI) used. The bismuth‐based quadruple therapy group exhibited a markedly superior success rate compared to the levofloxacin‐based triple therapy group when the latter was administered for 7 or 10 days (81.03% vs. 6.66%, *p* < 0.001, and 81.03% vs. 36.1%, *p* < 0.001, respectively). However, when the levofloxacin‐based triple therapy was extended to 14 days, its *H. pylori* eradication rate became comparable to that of the 10‐day bismuth‐based quadruple therapy (81.03% vs. 80%, *p* = 0.898). Eradication rates for both regimens were similar for patients aged ≥ 60, non‐Saudi, when using omeprazole and those treated with levofloxacin‐based triple therapy for 14 days.

**Conclusion:**

Quadruple treatment based on bismuth is superior to triple therapy based on levofloxacin for eradicating *H. pylori* in Saudi Arabia and should be used as a first‐line treatment. However, the 14‐day levofloxacin‐based triple treatment had an *H. pylori* eradication rate comparable to that of the 10‐day bismuth‐based quadruple therapy.

## 1 Introduction

*Helicobacter pylori* (*H. pylori*) infection is a highly prevalent global infection. Although developed countries have witnessed a decline in *H. pylori* prevalence owing to better sanitation and increased antibiotic usage, *H. pylori* remains highly prevalent (> 50%) in most other countries [1]. Infection is primarily responsible for peptic ulcer disease, mucosa‐associated lymphoid tissue lymphoma (MALT lymphoma), and gastric adenocarcinoma, and eradication is crucial for preventing or treating these diseases [2]. Traditionally, standard triple treatment, comprising a proton pump inhibitor (PPI) in conjunction with two antibiotics, has achieved an eradication rate exceeding 80% for *H. pylori* [3]. However, the efficacy of conventional treatments has been decreasing owing to rising global resistance to both clarithromycin and metronidazole [4]. In Saudi Arabia, the prevalence of clarithromycin‐resistant *H. pylori* ranges from 8.8% to 39.9%, whereas resistance to metronidazole ranges from 48.5% to 80%, indicating significant resistance rates [5, 6]. Therefore, unless antimicrobial susceptibility testing is performed, conventional triple therapy for 10–14 days is not advised for *H. pylori* treatment. Instead, bismuth‐based quadruple therapy for 10–14 days is now considered the preferred first‐ and second‐line treatment, as recommended by the Saudi *H. pylori* Working Group [6]. Numerous studies have demonstrated the effectiveness of levofloxacin‐containing triple therapy as second‐ and third‐line therapy [7, 8, 9, 10]. Levofloxacin‐containing triple therapy is also a successful first‐line therapy according to some studies [11, 12, 13]. Data on the effectiveness and duration of bismuth‐containing quadruple treatment with levofloxacin‐containing triple therapies are debatable. Some studies have shown that 1‐week quadruple treatments comprising bismuth and levofloxacin are effective first‐line treatments. Nevertheless, further research has shown that these regimens are unsuccessful when used as second‐line treatment [14, 15].

Considering the significance of local antibiotic‐resistance patterns, it is imperative to select treatment plans tailored to specific contexts. Consequently, our study aimed to investigate and compare the efficacy of levofloxacin‐based triple therapy with that of standard bismuth‐based quadruple therapy as the first‐line regimen for *H. pylori* eradication in Saudi Arabia.

## 2 Methods

### 2.1 Study Design and Patients

This retrospective study was conducted at the Dr. Sulaiman Al‐Habib Hospital in the eastern region of Saudi Arabia from January 2020 to August 2023. The study included adult patients diagnosed with *H. pylori* infection in gastroenterology clinics, identified by either histology or ^13^C‐urea breath test (UBT), who received treatment with either levofloxacin‐based triple therapy (Group I) or bismuth‐based quadruple therapy (Group II).

Patients with incomplete medical records, patients who received alternative treatment regimens, those who had previously attempted to eradicate *H. pylori*, those who had received treatment in the 4–6 weeks before UBT with an H_2_ receptor antagonist, PPI, or antibiotics, those who had stopped treatment early for any reason, and those who did not adhere to treatment were excluded. A ^13^C‐urea breath test was used to assess the rate of *H. pylori* eradication 4–8 weeks after therapy, and factors that might affect this rate of success were examined.

### 2.2 Therapeutic Regimens

Patients in Group I received levofloxacin‐based triple therapy: levofloxacin 500 mg once a day, amoxicillin 1 g twice a day, and PPI twice a day for 7, 10, or 14 days. Group II received Pylera (140 mg bismuth subcitrate potassium, 125 mg metronidazole, and 125 mg tetracycline hydrochloride) three capsules four times daily after meals and before sleep, plus PPI (omeprazole 20 mg, lansoprazole 30 mg, esomeprazole 40 mg, or pantoprazole 40 mg) twice daily before meals for 10 days. The PPI selection in both groups was determined by the prescribing physician.

### 2.3 Ethical Consideration

The Institutional Review Board of King Faisal University approved this research (KFU‐REC‐2024‐MAR‐ETHICS2147) and waived the requirement for patient consent owing to its retrospective nature. The study adhered to the Helsinki Declaration, anonymized the data, and conducted aggregate analysis.

### 2.4 Statistical Analysis

Statistical analyses were performed using SPSS (v 26.0; IBM Corporation, Armonk, NY, USA). Continuous variables were presented as mean ± standard deviation, whereas categorical variables were presented as numbers and percentages. The Chi‐squared test or Fisher's exact test was used to compare categorical variables, and the *t*‐test was used to compare continuous variables. Statistical significance was set at *p* < 0.05.

## 3 Results

### 3.1 Study Group Demographics

A total of 197 treatment‐naïve adults with *H. pylori* infection were included in our study. Eighty‐one patients received levofloxacin‐based triple therapy and 116 patients received bismuth‐based quadruple therapy. The relevant demographic characteristics of the study population are provided in Table 1. There were no significant differences between the two groups regarding age, sex, nationality, or type of PPI used.

**Table 1:** The basic characteristics of the two groups of patients.

	Group I (Levofloxacin‐based triple therapy)	Group II (Bismuth‐based quadruple Therapy)	Test	*p* value
	*n* = 81	*n* = 116		
Age (mean ± SD)	40.99 ± 13.1	41.2 ± 13.3	0.128^T^	0.89
Gender				
Male	40 (49.4%)	64 (55.2%)		
Female	41 (50.6%)	52 (44.8%)	0.64^C^	0.42
Nationality				
Saudi	77 (95.1%)	106 (91.4%)		
Non‐Saudi	4 (4.9%)	10 (8.6%)	0.98^C^	0.3
PPI				
Omeprazole	10 (12.3%)	15 (12.9%)		
Lansoprazole	11 (13.6%)	22 (19%)	5.056^C^	0.17
Pantoprazole	24 (29.6%)	45 (38.8%)		
Esomeprazole	36 (44.4%)	34 (29.3%)		
Treatment duration				
7 days	15 (18.5%)	0 (0%)		
10 days	36 (44.44%)	116 (100%)	—	—
14 days	30 (37%)	0 (0%)		

Abbreviations: PPI; proton pump inhibitor, T; *t*‐test, C; Chi‐square test.

### 3.2 Efficacy in *H. pylori* Eradication

**Figure 1:**
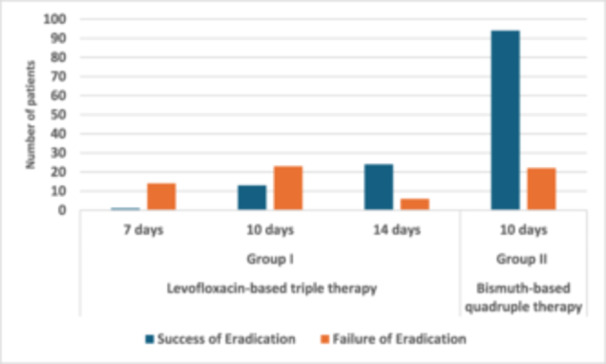
Efficacy in *Helicobacter pylori* eradication with both treatment regimens.

The effectiveness of both eradication regimens is shown in Figure 1. The overall eradication rate was 67% (132/197). The eradication rate in Group I was 6.66% (1/15) for those who received levofloxacin‐based triple therapy for 7 days, and 36.1% (13/36) for those who received it for 10 days, and 80% (24/30) for those who received it for 14 days. While the eradication rate was 81.03% (94/116) in Group II, which received bismuth‐based quadruple therapy (for 10 days). Therefore, when the levofloxacin‐based triple therapy is used for 7 or 10 days, the bismuth‐based quadruple therapy group had a significantly higher success rate than the levofloxacin‐based triple therapy group, but when it is used for 14 days, the *H. pylori* eradication rate is equivalent to that of the 10‐day bismuth‐based quadruple therapy.

### 3.3 Comparing the Studied Groups Categorized According to Their Outcomes

As shown in Table 2, there were no statistical differences in eradication rates between the two regimens in patients aged ≥ 60 years, in non‐Saudi patients, when omeprazole was used, and in patients treated with levofloxacin‐based triple therapy for 14 days.

**Table 2:** Comparison between the studied groups categorized according to their outcomes.

	Group I (Levofloxacin‐based triple therapy) (*N* = 81)		Group II (Bismuth‐based quadruple therapy) (*N* = 116)		Chi‐square/Fisher's exact test	*p* value
	Success	Failure	Success	Failure		
	*N* = 38	*N* = 43	*N* = 94	*N* = 22		
Age						
< 60 years ≥ 60 years	35/74 (47.3%) 3/7 (42.9%)	39/74 (52.7%) 4/7 (57.1%)	86/105 (81.9%) 8/11 (72.7%)	19/105 (18%) 3/11 (27.3%)	23.7 1.6	*p* < 0.001* *p* = 0.2
Gender						
Male Female	19/40 (47.5%) 19/41 (46.3%)	21/40 (52.5%) 22/41 (53.7%)	52/64 (81.3%) 42/52 (80.8%)	12/64 (18.8%) 10/52 (19.2%)	12.9 12.03	*p* < 0.001* *p* < 0.001*
Nationality						
Saudi Non‐Saudi	36/77 (46.8%) 2/4 (50%)	41/77 (53.2%) 2/4 (50%)	84/106 (79.2%) 10/10 (100%)	22/106 (20.8%) 0/10 (0%)	20.9 —	*p* < 0.001* —
Type of PPI						
Omeprazole Lansoprazole Pantoprazole Esomeprazole	5/10 (50%) 5/11 (45.5%) 10/24 (41.7%) 18/36 (50%)	5/10 (50%) 6/11 (54.5%) 14/24 (58.3%) 18/36 (50%)	13/15 (86.7%) 18/22 (81.8%) 33/45 (73.3%) 30/34 (88.2%)	2/15 (13.3%) 4/22 (18.1%) 12/45 (26.7%) 4/34 (11.8%)	2.48 4.59 6.68 11.86	*p* = 0.075 *p* = 0.03* *p* < 0.001* *p* < 0.001*
Treatment duration						
▪ 7 days for GI vs. 10 days for GII ▪ 10 days for GI vs. 10 days for GII ▪ 14 days for GI vs. 10 days for GII	1/15 (6.66%) 13/36 (36.1%) 24/30 (80%)	14/15 (93.3%) 23/36 (63.9%) 6/30 (20%)	94/116 (81%) 94/116 (81%) 94/116 (81%)	22/116 (19%) 22/116 (19%) 22/116 (19%)	10.58 26.6 0.017	*p* < 0.001* *p* < 0.001* *p* = 0.89

* Significant at *p* ≤ 0.05.

## 4 Discussion

*H. pylori* is a prevalent bacterial infection that can cause chronic gastritis, duodenal and stomach ulcers, gastric adenocarcinoma, and gastric MALT lymphoma [16]. It remains unknown how best to treat treatment‐naïve patients with *H. pylori* infection. Therefore, this study aimed to evaluate the effectiveness of triple therapy with levofloxacin and quadruple therapy with bismuth as the first‐line regimen in Saudi Arabia.

In the current study, the eradication rate in Group I (levofloxacin‐based triple therapy) was 6.66% for those who received levofloxacin‐based triple therapy for 7 days, 36.1% for those who received it for 10 days, and 80% for those who received it for 14 days, whereas that in Group II (bismuth‐based quadruple therapy) was 81.03%. So, the bismuth‐based quadruple treatment group had a significantly higher success rate than the levofloxacin‐based triple therapy group when the latter was given for 7 or 10 days. Nonetheless, when the levofloxacin‐based triple treatment was prolonged to 14 days, its *H. pylori* eradication rate became equivalent to that of the 10‐day bismuth‐based quadruple therapy.

According to a Turkish study, the eradication rate of bismuth quadruple therapy was greater than that of levofloxacin‐containing triple therapy (88.3% vs. 74.8%) [17]. In a different multicenter study conducted in Vietnam, the *H. pylori* eradication rates were 84.1% with a bismuth‐containing quadruple regimen and 77.4% with levofloxacin triple therapy (*p* < 0.05) [18].

A study from Saudi Arabia reported an eradication rate of 78.3% using bismuth quadruple therapy for 10 days in naïve patients or those previously treated with either standard triple therapy for 14 days or sequential therapy for 10 days [19]. A meta‐analysis examining *H. pylori* regimens revealed that quadruple therapy consisting of bismuth for 10–14 days resulted in an 85% eradication rate [20]. Trials conducted in Asia showed significantly higher eradication rates in individuals who received bismuth therapy as compared to trials conducted in Europe and the United States (82% vs. 74%) [21, 22, 23].

Several studies conducted worldwide evaluating the efficacy of bismuth‐based quadruple therapy for *H. pylori* eradication have shown varying results, none of which were identical. In 92.7% of treatment‐naïve individuals in an Italian study, *H. pylori* eradication was accomplished [12]. Delchier and colleagues treated *H. pylori*‐positive patients with bismuth‐based therapy in a multicenter, open‐label, single‐arm study conducted in France, Germany, Italy, and Spain. These authors have reported similar results. The eradication rates were 93.2% to 93.8%. In contrast, a 14‐day bismuth‐containing quadruple response to therapy was observed in 50% of the Lebanese patients with peptic ulcers [24, 25, 26]. The success of eliminating *H. pylori* infection is primarily dependent on *H. pylori* susceptibility to antibiotics included in the regimen. These disparate regimen results are likely attributable to the regional distribution of bacterial resistance [27].

One of the most noteworthy findings of our study was that within Group I, which consisted of levofloxacin‐based triple treatment, the eradication rate varied across different treatment intervals: 6.66% for 7 days, 36.1% for 10 days, and 80% for 14 days. Levofloxacin‐based first‐line therapy has been used in clinical practice since the year 2000. The total eradication rate across seven randomized controlled trials (RCTs) was 79.05% for the levofloxacin‐based regimen [28]. Other studies have reported *H. pylori* eradication rates ranging from 75% to 96% with levofloxacin‐based first‐line therapy [29, 30, 31, 32]. The 14‐day triple therapy with omeprazole, levofloxacin (500 mg daily), and amoxicillin had a success rate of 75% [33].

In a Kosovo trial, 105 patients were randomly assigned to receive levofloxacin‐based regimens consisting of omeprazole, levofloxacin (500 mg daily), and amoxicillin for either 7 or 10 days; and 86.2% and 93.6% of *H. pylori* infections were eradicated [34]. Combining PPI, amoxicillin, and levofloxacin has been shown in previous research to be the most effective way to eliminate *H. pylori* (~90% success rate) [35, 36].

Wu and colleagues discovered that levofloxacin‐based triple therapy had an *H. pylori* eradication rate of 67.6%, which was lower than that reported in previous studies [37]. A meta‐analysis revealed significant variation in the response to triple therapy with levofloxacin. The clear causes of high heterogeneity were not apparent. The study population was the primary distinction among studies. Levofloxacin‐resistant *H. pylori* strains are becoming increasingly common worldwide, particularly in countries with a high quinolone intake. Resistance reduces the effectiveness of levofloxacin‐based regimens in eliminating *H. pylori* [38]. When fluoroquinolone resistance is high, levofloxacin‐based triple therapy becomes ineffective. In Italy, the eradication rate has dropped dramatically from 75% in levofloxacin‐susceptible patients to 33.3% in patients with levofloxacin‐resistant strains [39]. A prospective study in Saudi Arabia in 2015 found that *H. pylori* resistance to metronidazole, clarithromycin, amoxicillin, levofloxacin, and tetracycline was 48.5%, 23.3%, 14.8%, 11.1%, and 2.3%, respectively [40]. In a trial conducted in Saudi Arabia by Alsohaibani and colleagues, levofloxacin‐based triple treatment was found to be less effective as rescue therapy (36.36%) [41].

Because of increased rates of levofloxacin resistance, levofloxacin should not be used for therapy unless the *H. pylori* strain is known to be responsive to it or the population's levofloxacin resistance rate is < 15% [42, 43]. Levofloxacin‐based first‐line therapy may be a viable option for patients in places where quinolone resistance is low but clarithromycin resistance is significant [18, 28]. When first‐line clarithromycin therapy fails, the ACG Clinical Guidelines for the Management of *H. pylori* infection recommend bismuth quadruple therapy or levofloxacin salvage regimens [3].

In the current study, all patients in Group II who underwent bismuth triple therapy had a 10‐day treatment duration. In a study that examined 6 RCTs and 4763 patients, there was no significant difference in the success rates of bismuth‐containing quadruple regimens at 7, 10, or 14 days [44]. There were no significant differences in efficacy between bismuth quadruple treatment regimens administered for 10 or 14 days [45].

In Group I, which received levofloxacin triple therapy, there was a substantial difference in the treatment response, with a longer duration of therapy (14 days). This is supported by a study conducted in Turkey, which showed that levofloxacin triple therapy worked much better when administered for a longer period as a first‐line treatment (72% with a 14‐day regimen vs. 34% with a 7‐day regimen) [46]. This is also consistent with guidelines that prescribe longer (14 days) treatments with all antibiotic regimens for *H. pylori* [47, 48].

We found no statistical differences in eradication rates between the two regimens in patients aged ≥ 60 years and non‐Saudi patients. Previous studies have shown that the eradication rate of *H. pylori* treatment regimens is not related to age [49, 50]. While others have revealed that age influences *H. pylori* eradication in both developed and developing nations [51, 52], and the results of a study conducted in Pakistan indicated that there has been a substantial rise in the incidence of failure among both young and elderly individuals. Conversely, the middle‐aged group showed a higher eradication [53].

In addition, there was no statistical difference in the eradication rate across patients for either regimen when omeprazole was used. In contrast, Vergara and colleagues conducted a MEDLINE search for a meta‐analysis of 14 studies comparing the efficacy of various PPIs in triple therapy, which yielded comparable results [54]. However, another study discovered that esomeprazole and rabeprazole had a slight advantage over first‐generation PPIs in terms of overall *H. pylori* eradication rates [55].

Although triple therapy based on traditional PPIs is successful, prolonged acid suppression treatments are attracting increasing attention. New regimens for *H. pylori* eradication include potassium‐competitive acid blocker (P‐CAB) triple and dual treatment. According to a recent meta‐analysis and systematic review, triple therapy using vonoprazan, an acid blocker that competes with potassium, worked better than triple therapy using traditional PPIs. This makes it an even better first‐line treatment [56].

The most interesting finding in our study was that the 14‐day levofloxacin‐based triple therapy achieved an *H. pylori* eradication rate comparable to that of the 10‐day bismuth‐based quadruple therapy. According to a study from Turkey, standard bismuth‐based quadruple therapy was better at eliminating *H. pylori* infections (> 90%) than levofloxacin‐containing triple therapy (79.2%) [57]. Another study from Vietnam concluded that the initial four‐drug regimen with bismuth is more effective at eradicating *H. pylori* than three‐drug treatment with levofloxacin as the first‐line treatment; however, the patient's compliance rate was lower than that of the levofloxacin‐based three‐drug regimen [18]. This finding highlights that the longer, but one drug less, triple therapy may be more readily accepted by patients, considering the associated treatment costs and potential side effects.

To optimally treat *H. pylori* infections, local antibiotic resistance and eradication patterns must be carefully considered. Thus, quadruple therapy may play a larger role in the eradication of *H. pylori* [47]. Owing to the high prevalence of resistance to clarithromycin and metronidazole, the Saudi *H. pylori* Working Group concluded that antimicrobial susceptibility testing is necessary before administering conventional triple therapy for 10–14 days to treat *H. pylori*. According to the available data, bismuth‐based quadruple treatment for 10–14 days is the most effective first‐ and second‐line therapy in Saudi Arabia [58].

A limitation of the present study was the lack of regional antibiotic resistance patterns. Because antibiotic resistance rates vary among regions, the findings of this study cannot be applied internationally. Other limitations include its retrospective nature with the small number of patients in each group. Other restrictions included the absence of information regarding smoking and alcohol consumption, adverse effects of the two regimens, and clinical presentation or endoscopic findings of our patients.

## 5 Conclusion

Standard bismuth‐based quadruple therapy for *H. pylori* has achieved satisfactory eradication rates. Levofloxacin‐based triple treatment has a lower efficacy in *H. pylori* eradication compared to bismuth‐based quadruple therapy when administered for short periods (7 or 10 days). The optimal duration of levofloxacin‐based triple therapy was 14 days, resulting in a higher H. pylori eradication rate equivalent to that of the 10‐day bismuth‐based quadruple therapy. This highlights that, for successful *H. pylori* eradication, treatment tactics must be tailored to local antibiotic resistance trends. Future large‐scale randomized studies are warranted to determine the most effective treatment method for *H. pylori* infection in Saudi Arabia.

## Author Contributions

**Abdulrhman Khaled Al Abdulqader:** conceptualization, supervision. **Turki Abdullah Alamri:** visualization. **Mahdi Abdullah Alhamad:** data curation. **Somaia Shehab El‐Deen:** analysis of the results, supervision, writing – original draft. **Abdallah Essa:** methodology, writing – review and editing. **Raed Abdullah Alfayez:** data curation. **Baqer Mohammed Albaqshi:** data curation. **Adnan Salah Almajed:** data curation. **Mohammed Yousef Alhassan:** data curation. **Ali Essa:** writing – original draft, writing – review and editing. **Ahmed Abdullah Albadrani:** visualization. **Omar Alomair:** methodology. **Bashaeer Abdullh Al Jalal:** writing – review and editing. **Mohammed Yousef Almulhim:** methodology. **Abdullah Alotaibi:** visualization. **Ehab Darwish:** writing – original draft, supervision. All authors have read and approved the final version of the manuscript.

## Conflicts of Interest

The authors declare no conflicts of interest.

## Transparency Statement

The lead author Abdulrhman Khaled Al Abdulqader affirms that this manuscript is an honest, accurate, and transparent account of the study being reported; that no important aspects of the study have been omitted; and that any discrepancies from the study as planned (and, if relevant, registered) have been explained.

## Data Availability

The data of this study are available from the first author upon reasonable request.
